# Rapid and Visual Detection of *Trichinella* Spp. Using a Lateral Flow Strip-Based Recombinase Polymerase Amplification (LF-RPA) Assay

**DOI:** 10.3389/fcimb.2019.00001

**Published:** 2019-01-21

**Authors:** Ting-Ting Li, Jin-Lei Wang, Nian-Zhang Zhang, Wen-Hui Li, Hong-Bin Yan, Li Li, Wan-Zhong Jia, Bao-Quan Fu

**Affiliations:** ^1^State Key Laboratory of Veterinary Etiological Biology, Key Laboratory of Veterinary Parasitology of Gansu Province, Lanzhou Veterinary Research Institute, Chinese Academy of Agricultural Sciences, Lanzhou, China; ^2^Jiangsu Co-innovation Center for the Prevention and Control of Important Animal Infectious Diseases and Zoonoses, Yangzhou University College of Veterinary Medicine, Yangzhou, China

**Keywords:** *Trichinella*, recombinase polymerase amplification, diagnostics, lateral flow strip, rapid test

## Abstract

*Trichinella* spp., are amongst the most widespread parasitic nematodes, primarily live in the muscles of a wide range of vertebrate animals and humans. Human infection occurs by ingestion of raw or undercooked meat containing *Trichinella* larvae. Accurate diagnosis of *Trichinella* spp. infection in domestic animals is crucial for the effective prevention and control of human trichinellosis. In the present study, a simple, rapid and accurate diagnostic assay was developed combining recombinase polymerase amplification and a lateral flow strip (LF-RPA) to detect *Trichinella* spp. infection. The LF-RPA assay targets *Trichinella* spp. mitochondrial small-subunit ribosomal RNA (*rrnS*) gene and can detect as low as 100 fg DNA of *Trichinella* strains, which was approximately 10 times more sensitive than a conventional PCR assay. The LF-RPA assay can be performed within 10–25 min, at a wide range of temperatures (25–45°C) and showed no cross-reactivity with DNA of other parasites and related host species of *Trichinella*. The performance of the LF-RPA assay in the presence of high concentration of PCR inhibitor was better than that of a conventional PCR assay. Results obtained by LF-RPA assay for the detection of experimentally infected mice were comparable to the results obtained by using a conventional PCR, achieving 100% specificity and high sensitivity. These results present the developed LF-RPA assay as a new simple, specific, sensitive, rapid and convenient method for the detection of *Trichinella* infection in domestic animals.

## Introduction

Trichinellosis, one of the most important food-borne parasitic zoonosis, is caused by consumption of raw or undercooked meat containing infective larvae of the nematodes of the genus *Trichinella* (Cui and Wang, 2011; Murrell and Pozio, 2011; Pozio, 2015; Murrell, 2016; Zhang et al., 2018). *Trichinella* can infect a wide range of vertebrates including humans. It is estimated that approximately 11 million people may be infected with this parasite (Kurdova-Mintcheva et al., 2009). Outbreaks of trichinellosis in humans have been documented in different areas of the world (Kurdova-Mintcheva et al., 2009; Dubinský et al., 2016; Bai et al., 2017; Ng-Nguyen et al., 2017; Rostami et al., 2017; Turiac et al., 2017). However, control and prevention of this parasite has been difficult due to interconexions among epidemiological cycles and the lack of effective parasite surveillance system in many countries (Gottstein et al., 2009; Wang et al., 2017). The development of a simple, rapid and accurate diagnostic method for the detection of *Trichinella* infection in domestic animals is important for effective control and surveillance of this disease.

Currently, the clinical diagnosis of trichinellosis is very difficult because most *Trichinella* infections are asymptomatic or with non-specific clinical manifestations (Gottstein et al., 2009; Shimoni and Froom, 2015). Microscopic examination and serological assays are used to diagnosis of *Trichinella* infection in domestic or wild boars (Gottstein et al., 2009; Cuttell et al., 2012; Fu et al., 2013; Lin et al., 2013; Shimoni and Froom, 2015; Sun et al., 2015). Microscopic examinations are routinely used for the detection of *Trichinella* larvae in muscle tissues at slaughtering. However, the microscopic examination is labor-intensive, low sensitive, time-consuming, and also requires the use of microscope and a trained personnel (Gottstein et al., 2009; Shimoni and Froom, 2015). Serological assays have been useful for epidemiological studies and large-scale disease surveillance, but these immunologic diagnostic methods cannot replace the direct detection methods used for meat inspection due to the potential cross-reactivity with other parasites (Gottstein et al., 2009; Cuttell et al., 2014; Shimoni and Froom, 2015; Wang et al., 2017). The PCR based diagnostic methods such as conventional PCR, real-time PCR, and multi-PCR methods have been developed to detect *Trichinella* DNA (Lin et al., 2013; Shimoni and Froom, 2015). Although PCR-based assays are highly sensitive and can detect low parasite burdens, they require expensive instruments and a trained technician, making the use of PCR based-methods difficult in resource-limited settings (Gottstein et al., 2009; Cuttell et al., 2012; Lin et al., 2013; Shimoni and Froom, 2015). Therefore, a rapid, sensitive, specific, and field-applicable diagnostic method is clearly desired to improve the effectiveness of *Trichinella* control and surveillance programs.

The recombinase polymerase amplification (RPA), an isothermal DNA amplification technology, has been developed for the diagnosis of several pathogens (James and Macdonald, 2015; Daher et al., 2016). This RPA technique does not require the use of thermal cycling apparatus to denature DNA template, but instead utilizes recombinase-primers to scan for homologous sequences in a DNA template and facilitates DNA strand exchange at cognate sites (James and Macdonald, 2015; Daher et al., 2016). The RPA assay can be performed rapidly and results can be obtained in <20min and at temperature range between 25 and 45°C (James and Macdonald, 2015; Daher et al., 2016). Additionally, RPA DNA amplified products can be detected by simple lateral flow (LF) strips and the results can be easily read without any specialized equipment, which provides convenient diagnostic assay for the detection of *Trichinella* infection in resource-limited settings (James and Macdonald, 2015; Daher et al., 2016).

In the present study, a rapid and simple LF-RPA diagnostic method was developed to test for *Trichinella* spp. infection in domestic animals. The sensitivity and specificity of this assay were investigated in comparison with a conventional PCR assay. In addition, the effectiveness of LF-RPA was evaluated in the presence of PCR inhibitors of the muscle and in samples obtained from experimentally infected mice.

## Materials and Methods

### Primers and Probe Design

The mitochondrial small-subunit ribosomal RNA (*rrnS*) gene was selected as target for designing RPA primers because this part of the mitochondrial DNA is highly conserved among all known *Trichinella* species and genotypes (Cuttell et al., 2012). Primers and a probe specific to the *rrnS* gene of *Trichinella* were designed according to the instruction manual of TwistDX. The RPA PCR primers and probe were analyzed by Oligo primer analysis software for duplex formation, hairpin formation, and similar biophysical properties and blasted against the National Center for Biotechnology Information nucleotide database to validate the lack of sequence homology with other related species. Further, the repeatability and specificity of the designed primer candidates were evaluated by the RPA nfo kit (TwistDx, UK). The RPA amplicon was detected on a 2.5% agarose gel to identify the optimal primers. One primer pair that resulted in a 195 bp amplicon was selected. In order to be visualized by lateral flow detection system, a biotin was added at the 5′ end of the reverse primer. A specific internal probe was designed to add a 5′ fluorescein FAM, an abasic furan (dSpacer) and a C3 spacer (SpC3) on the 3′ end (Table 1). For conventional PCR, a pair of primers specific to the *rrnS* gene, T-rrnS-F (5′-CATGGTTAGGTGAGATATTGCCTGC-3′) and T-rrnS-R (5′-GGTCCTCCTTCCAGAAGATCTACTTTG-3′) were used to detect *Trichinella* DNA as described previously (Cuttell et al., 2012).

**Table 1:** Primers and probe used in the present study for LF-RPA.

**Primer name**	**Sequence (5′-3′)**
RPA-F	CATGGTTAGGTGAGATATTGCCTGCAAATA
RPA-R	GGTCCTCCTTCCAGAAGATCTACTTTGTTA
Lateral flow reverse primer	biotin-GGTCCTCCTTCCAGAAGATCTACTTTGTTA
	FAM-CCCACTAAATTCCTTATGCACATATTGCCC-dSp
Lateral flow probe	acer-TCACCCTCATAAGAG-C3Spacer

### Sources of *Trichinella* and Tissue Materials

The Chinese *Trichinella spiralis* Henan isolate (ISS534, T1), *T. spiralis* Yunan isolate, *T. spiralis* Xi'an isolate, *T. spiralis* Tibet isolate, and three *Trichinella pseudospiralis* strain (ISS141, T4), *T. pseudospiralis* (ISS470, T4), and *T. pseudospiralis* Russin were maintained in specific pathogen-free male Kunming mice in our laboratory as described previously (Fu et al., 2009). The DNAs of *T. nativa* (ISS10, T2); *T. britovi* (SS120, T3); *T. murrelli* (ISS417, T5); *T. nelsoni* (ISS37, T7); *T. papuae* (ISS1980, T10); *T. zimbabwensis* (ISS1029, T11); *T. patagoniensis* (ISS2496, T12); and *Trichinella* genotypes T6 (ISS34), T8 (ISS272), and T9 (ISS409) were kindly provided by Ming-Yuan Liu. Genomic DNA from *Toxoplasma gondii, Toxocara canis, Cooperia oncophor, Fasciola hepatica, Gongylonema pulchrum, Taenia solium, Echinococcus granulosus*, and *Echinococcus multilocularis* were obtained from stocks in our institute. In addition, genomic DNA from related hosts of *Trichinella*, including mouse (*Mus musculus*) and wild boar (*Sus scrofa*), domestic pig (*Sus scrofa domesticus*), human (*Homo sapiens*), dog (*Canis lupus familiaris*), horse (*Equus caballus*), rat (*Rattus norvegicus*), cow (*Bos taurus*) cat (*Felis domestica*), sheep (*Ovis aries*) and rabbit (*Oryctolagus cuniculus*) were also available in our institute.

### Experimental Infection

*Trichinella spiralis* isolate obtained from domestic pigs in Henan Province was used to experimentally infect 8-week-old, SPF male Kunming mice (Liu et al., 2016). Briefly, ten Kunming mice were divided equally into two groups (naïve control group and *T. spiralis* infected group). For the infected group, five mice were orally inoculated with 300 muscle larvae of *T. spiralis* per mouse. Mice were sacrificed on days 35 post-inoculation and the mice not inoculated with *T. spiralis* were used as the negative control. Fifty milligram of mice tissues including tongue, diaphragm, and abdominal muscle collected from each mouse were used for DNA extraction. Before DNA extraction, all the tissues were compressed and detected by microscope to ensure the presence of *T. spiralis* larvae.

### Spiked Muscle Sample Preparation

*T. spiralis* and *T. pseudospiralis* larvae were used to spike swine muscle samples that were negative for *Trichinella* based on microscopic detection. *T. spiralis* and *T. pseudospiralis* samples prepared by individually observing muscle larvae under a dissecting microscope were transferred to a 5 ml flat-bottomed plastic centrifuge tube by a micropipette. Larvae were visually counted, ensuring that *T. spiralis* or *T. pseudospiralis* larvae were transferred. One gram of muscle tissue from swine was finely minced into small pieces and spiked with one *T. spiralis* larva (Lin et al., 2013). Then, DNA was extracted for further assessment of the LF-RPA assays.

### DNA Extraction

Pure genomic DNA from 500 *T. spiralis* larvae or *T. pseudospiralis* as well as genomic DNA from tissues samples of experimentally infected mice using a TIANamp Genomic DNA Kit (Tiangen Biotech, China) according to the manufacturer's instructions. For genomic DNA from spiked muscle samples, TIANamp Genomic DNA Kit was used with some modifications. Briefly, the muscles were minced into small pieces, and GA lysis buffer was added to 1 g of *Trichinella*-spiked muscle to a total volume of 3 ml and then 200 μL proteinase K were added. Samples were incubated overnight at 56°C to ensure complete homogenization. Then, 400 μl of crude lysate was transferred to a 2 ml microfuge tube and 400 μl buffer GB added before incubation at 70°C for 10 min. The samples were then centrifuged for 1 min at 13,000 g to pellet any remaining cellular debris and about 780 μl supernatant removed to a clean 2 ml microfuge tube containing 400 μl ethanol. After being vortexed thoroughly, the samples were transferred twice to the Spin Column CB3 each with about 590 μl, and the remaining steps were followed as per the kit protocol. Genomic DNA was eluted in 100 μl of distilled water and the quantity and quality was tested by a NanoDrop spectrophotometry (Thermo Scientific, USA).

### RPA Reaction and Lateral Flow Reading

A 50 μl RPA reaction mixture was performed using a TwistAmp nfo kit (TwistDX Ltd., Cambridge, UK) according to the manufacturer's instructions. Briefly, each RPA reaction mixture included 29.5 μl TwistAmp rehydration buffer, 0.6 μl RPA probe (10 μM), 2.1 μl each RPA primer (10 μM), 11.2 μl distilled water, and 2 μl genomic DNA template. A total of 47.5 μl of master mix were pipetted into a 200 μl reaction tube supplied with a dried enzyme pellet. Finally, a total of 2.5 μl magnesium acetate was added to the lid of the tube and the tube was briefly centrifuged to mix the magnesium acetate into the reaction mixture to start RPA reaction. The tube was incubated at 37°C for 20 min with constant shaking at 180 rpm in a thermos shaker incubator. After amplification, the RPA amplicon was purified with a PCR Purification Kit (Tiangen Biotech, China) and then examined on a 2.5% agarose gel. For the detection of RPA amplicon directly, 2 μl RPA reaction mixtures were mixed with 198 μl dipstick assay buffer. Then, 10 μl of the diluted sample was transferred to the sample pad of a Genline Hybridetect-1 lateral flow strip (Milenia Biotec, Germany), and then, the strip end was vertically placed into 200 μl of running buffer and incubated 5 min at room temperature. DNA amplification was observed with the naked eye as indicated by the appearance of the test line. Appearance of a control line in the upper part of the strip confirms the successful test run of the system.

### Evaluation of Lateral Flow RPA Conditions

The optimal RPA reaction time and temperature were determined by examining different times (0–25 min) and various temperature settings ranging from 20 to 50°C. The sensitivity of the RPA reaction was evaluated using 10-fold serial dilutions of *T. spiralis* or *T. pseudospiralis* DNA, ranging from 1 ng to 10 fg per reaction. For specificity testing, 1 ng genomic DNA of *T. spiralis* and at least 10 ng genomic DNA templates from other parasites and host species were utilized in a 50 μl RPA reaction mixture.

Various substances in the muscle are known to inhibit the enzymatic nucleic acid amplification (Al-Soud and Radstrom, 2001; Garcia et al., 2002). Some inhibitors may be introduced during the sample collection or processing, especially in resource-limited settings. To test the effects of inhibitors on the assay performance, 1 ng of *T. spiralis* or *T. pseudospiralis* genome was utilized in a typical 50 μl RPA reaction mixture which included a various volume of muscle penetrating fluid from swine (0% [negative control with distilled water], 4, 8, 12, 16, and 20%). For comparison with conventional PCR assay (Cuttell et al., 2012), the same percentages of the pig muscle penetrating fluid volume were added to the conventional PCR reaction mixture. The amplification reaction was performed in 50 μl containing 25 μl Premix Taq (TaKaRa, Dalian, China), T-rrnS-F (10 μM) 2 μl, T-rrnS-R (10 μM) 2 μl and corresponding DNA or penetrating fluid volume or distilled water. The reaction conditions were: 94°C for 5 min, then 30 cycles of 30 s at 94°C, 30 s at 53°C and 30 s at 72°C, and a final extension at 72°C for 5 min. Distilled water was used as negative control.

## Results

### Evaluation of Amplification Time and Temperature

**Figure 1:**
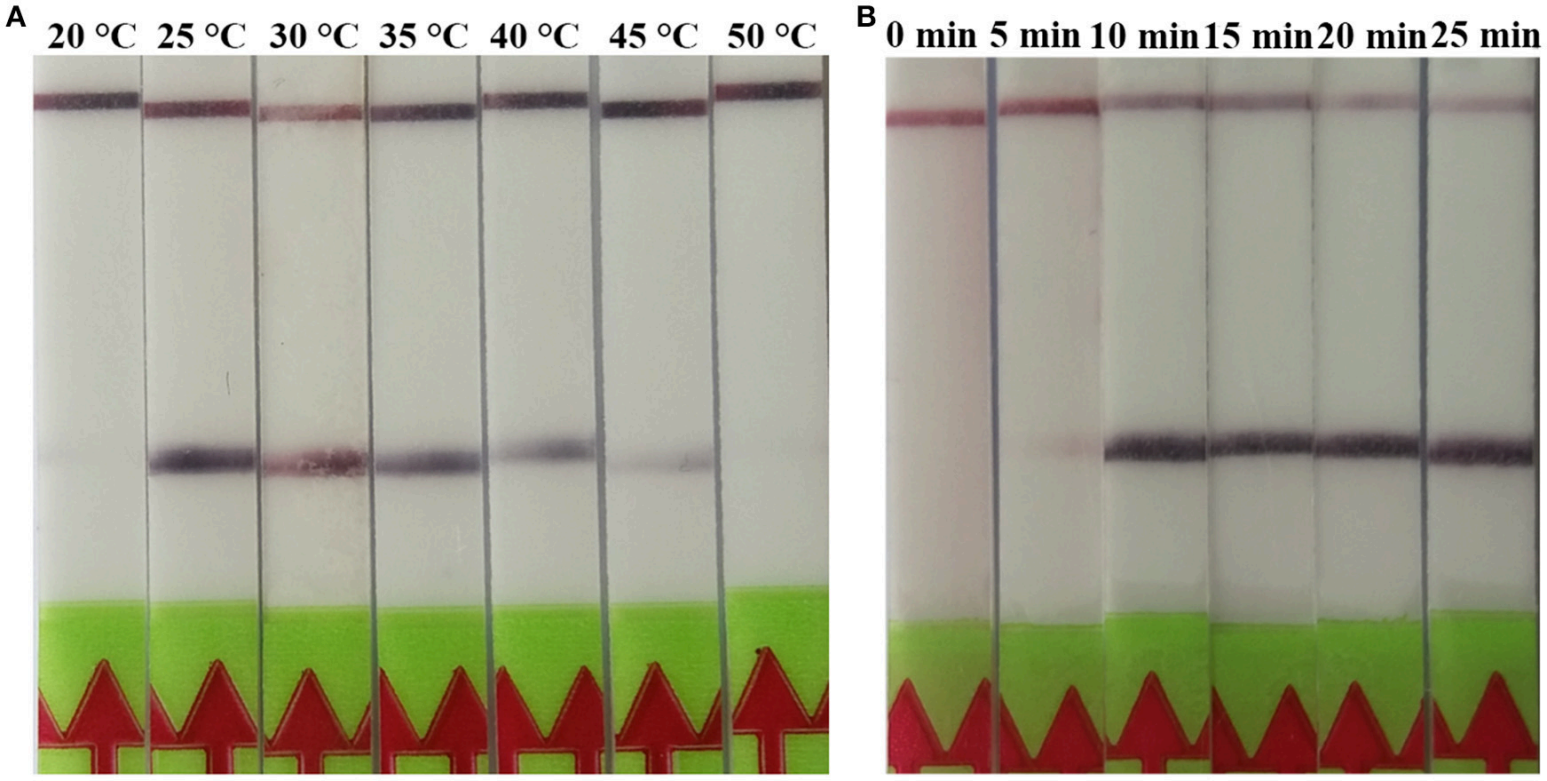
Evaluation of the amplification temperatures and amplification times on the LF-RPA assay. **(A)** The LF-RPA worked well in a wide range of amplification temperature from 25 to 45°C. **(B)** After 10min of isothermal amplification reaction, the positive reaction was visible on the test strip.

The RPA reaction temperature was tested at wide range of temperatures from 20 to 50°C. The results showed that the RPA reaction performs well from 25 to 45°C after 5 min incubation on the lateral flow strip, however, the brightness of the test line became weaker as the temperature fluctuates out of this range (Figure 1A). Our results indicated that RPA reaction appeared to be not sensitive to the reaction temperature. The RPA amplification was achieved at time ranging from 0 min to 25 min at 37 °C. The results showed that a faint test line could be detected when the amplification time was 5 min. As the amplified time increased to 10 min, the test line becomes brighter (Figure 1B).

### Analytical Specificity of LF-RPA

**Figure 2:**
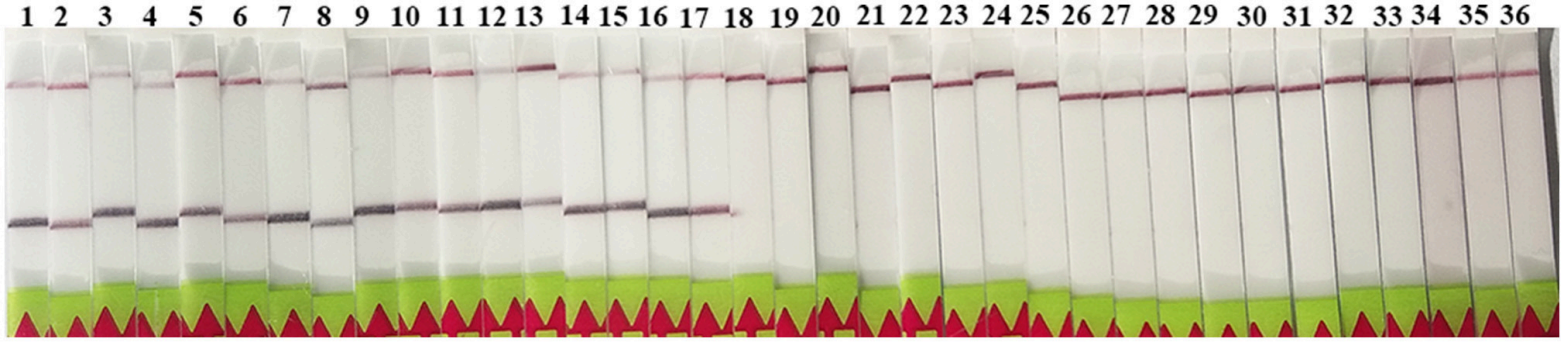
Specificity of the LF-RPA assay. Line 1: *T. spiralis* Henan strain (ISS534, T1); line 2: *T. nativa* (ISS10, T2); line 3: *T. britovi* (SS120, T3); line 4: *T. pseudospiralis* (ISS141, T4); line 5: *T. murrelli* (ISS417, T5); line 6: *Trichinella* genotypes T6 (ISS34); line 7: *T. nelsoni* (ISS37, T7); line 8: *Trichinella* genotypes T8 (ISS272); line 9: *Trichinella* genotypes T9 (ISS409); line 10: *T. papuae* (ISS1980, T10); line 11: *T. zimbabwensis* (ISS1029, T11); line 12: *T. patagoniensis* (ISS2496, T12); line 13: *T. spiralis* Xi'an isolate; line 14: *T. spiralis* Yunnan isolate; line 15: *T. spiralis* Tibet isolate; line 16: *T. pseudospiralis* (ISS470, T4); line 17: *T. pseudospiralis* Russin; line 18: *Toxoplasma gondii*; line 19: *Cooperia oncophor*; line 20: *Fasciola hepatica*; line 21: *Gongylonema pulchrum*; line 22: *Taenia solium*; line 23: *Echinococcus granulosus*; line 24: *Echinococcus multilocularis*; line 25: *Toxocara canis*; line 26: mice (*Mus musculus*); line 27: wild boar (*Sus scrofa*); line 28: domestic pig (*Sus scrofa domesticus*); line 29: human (*Homo sapiens*); line 30: dog (*Canis lupus familiaris*); line 31: horse (*Equus caballus*); line 32: rat (*Rattus norvegicus*); line 33: cow (*Bos taurus*); line 34: cat (*Felis domestica*); line 35: sheep (*Ovis aries*); and line 36: rabbit (*Oryctolagus cuniculus*).

The LF-RPA primers were selected to target to region of the *rrnS* gene, which was conserved among all known *Trichinella* species (Cuttell et al., 2012). Our results showed that the primers detected all known 12 species and genotypes of *Trichinella* as revealed by the presence of a clearly visible band on the lateral flow strip, suggesting that the primers have the ability to amplify a wide range of *Trichinella* species and genotypes (Figure 2). In addition, there was no cross-reactivity with DNA samples from other parasites or related hosts of *Trichinella* tested in the present study (Figure 2).

### Analytical Sensitivity of LF-RPA

**Figure 3:**
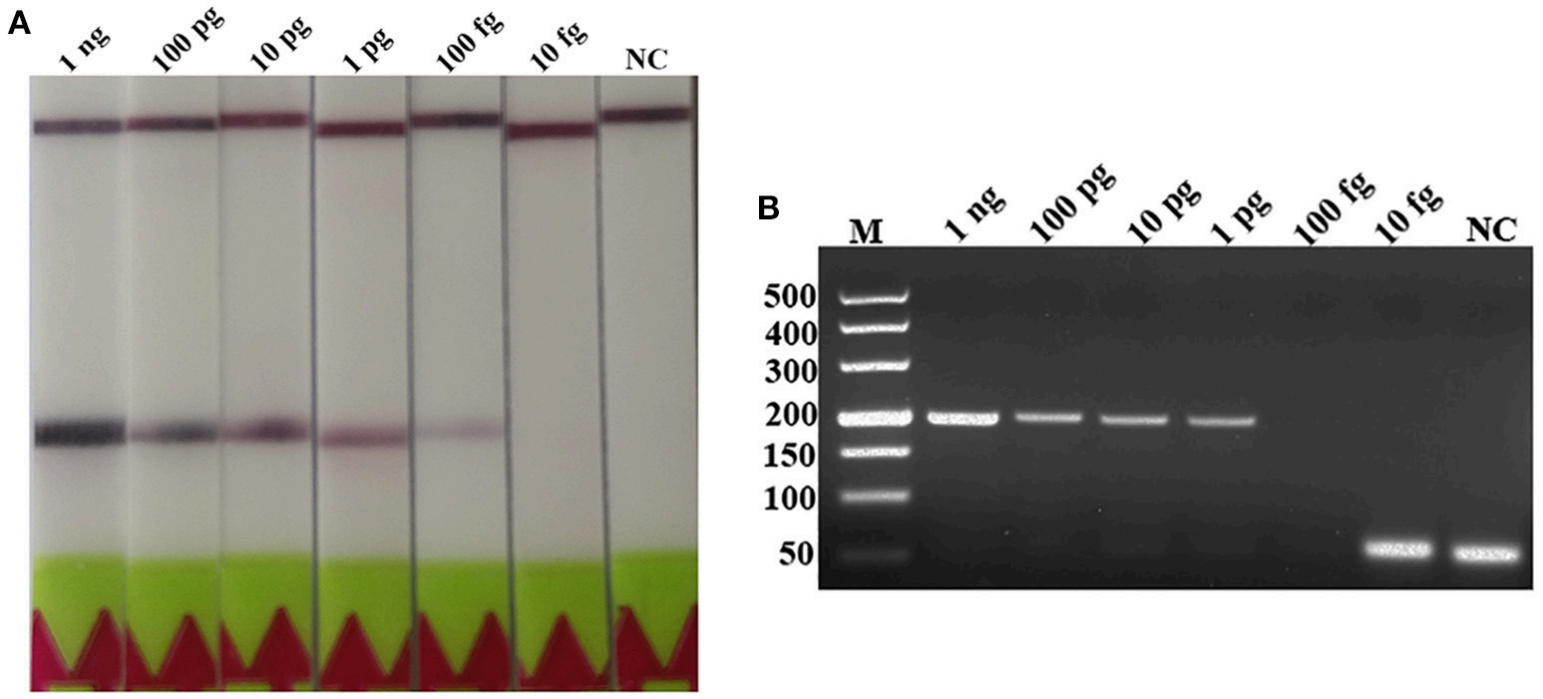
Sensitivities of the LF-RPA assay and conventional PCR assay for the detection of isolated genomic DNA from *T. spiralis*. Ten-fold serial dilutions of *T. spiralis* genomic DNA (1 ng/reaction to 10 fg/reaction) were evaluated by LF-RPA **(A)** and conventional PCR assay detected by agarose gel electrophoresis **(B)**. Lower limit of detection can be seen at 100 fg and 1 pg of *T. spiralis* DNA by LF-RPA assay and conventional PCR assay, respectively. NC, Negative Control.

**Figure 4:**
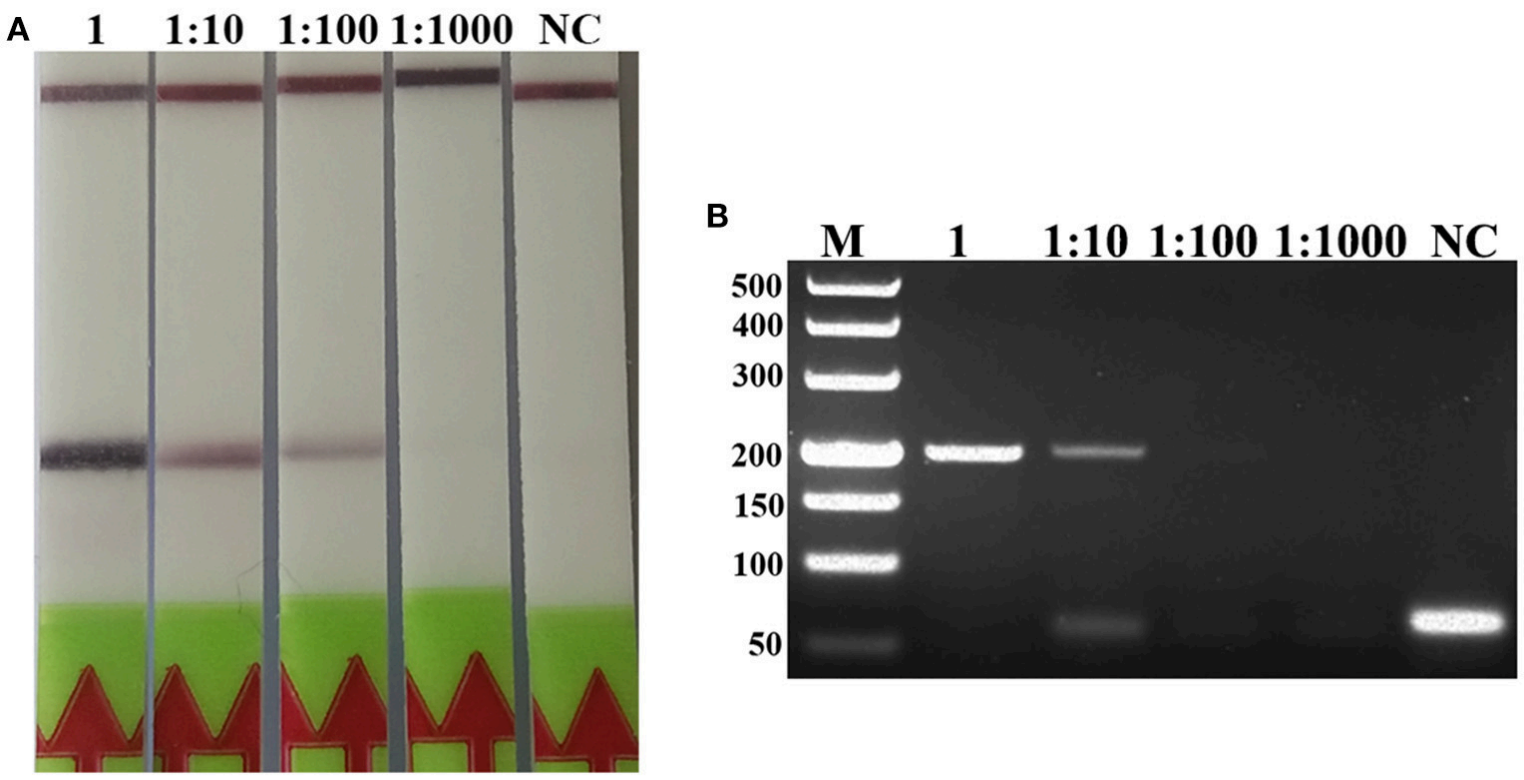
Sensitivities of the LF-RPA assay and conventional PCR assay for detecting *T. spiralis* DNA extracted from swine muscle. One-gram from each muscle sample was spiked with one *T. spiralis* larvae and 10-fold serial dilutions of this DNA were evaluated by LF-RPA **(A)** and conventional PCR assay detected by agarose gel electrophoresis **(B)**. Lower limit of detection can be seen at 1:100 and 1: 10 dilutions of this DNA by LF-RPA assay and conventional PCR assay, respectively. NC, Negative Control.

A serial dilutions of *T. spiralis* or *T. pseudospiralis* DNA, ranging from 1 ng to 10 fg per reaction were used to evaluate the detection threshold using lateral flow strip. Results showed that the RPA assay was highly sensitive with a detection limit of 100 fg, corresponding to approximately 0.001 larvae (Figure 3A and Figure S1A). The conventional PCR assay was less sensitive than RPA assay, which can only detect 1 pg DNA per reaction (Figure 3B and Figure S1B). These two assays have the ability to detect one larva in 1 g swine muscle. However, 100-fold diluted DNA could be detected only by the LF-RPA assay (Figure 4 and Figure S2). The results obtained from three independently experiments with three replicates per experiment showed that the RPA assay was almost 10 times more sensitive than the conventional PCR assay.

### The Effect of Inhibitors

**Figure 5:**
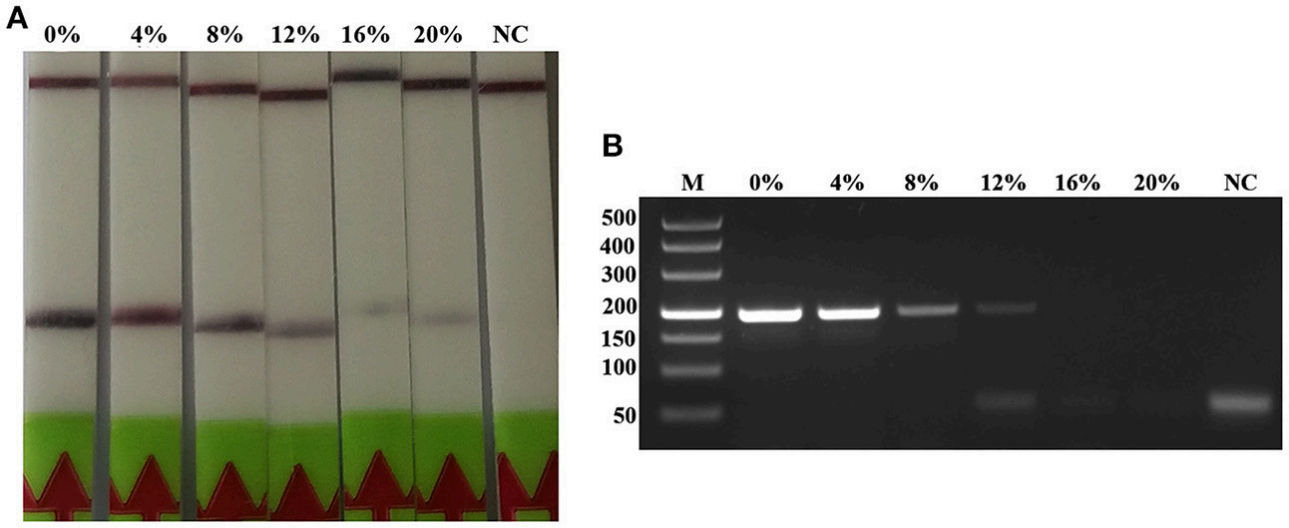
Effects of inhibitors on the performance of the LF-RPA assay and conventional PCR assay for detecting the DNA from *T. spiralis*. Different percentage of muscle penetrating fluid in the reaction mixture were evaluated using LF-RPA assay **(A)** and conventional PCR assay **(B)**. LF-RPA assay performed better than conventional PCR assay in the presence of potential inhibitors of the amplification reaction. NC, Negative Control.

The detection of *Trichinella* is usually performed directly with muscle samples. Therefore, effect of the potential inhibitory substances in the muscle was tested by addition of increasing volumes of the swine muscle penetrating fluid in the RPA reaction mixture or the conventional PCR reaction mixture. Each reaction was adjusted with distilled water to the total required volume. Results showed that although both conventional PCR assay and LF-RPA assay can work well in the presence of (< 16%) muscle penetrating fluid (Figure 5 and Figure S3), LF-RPA assay can work better than conventional PCR assay in the presence of 16% muscle penetrating fluid. These results indicate that LF-RPA system is less likely to be affected by the inhibitors present in the muscles compared to the conventional PCR system.

### Evaluation of LF-RPA for Experimentally Infected Samples

**Figure 6:**
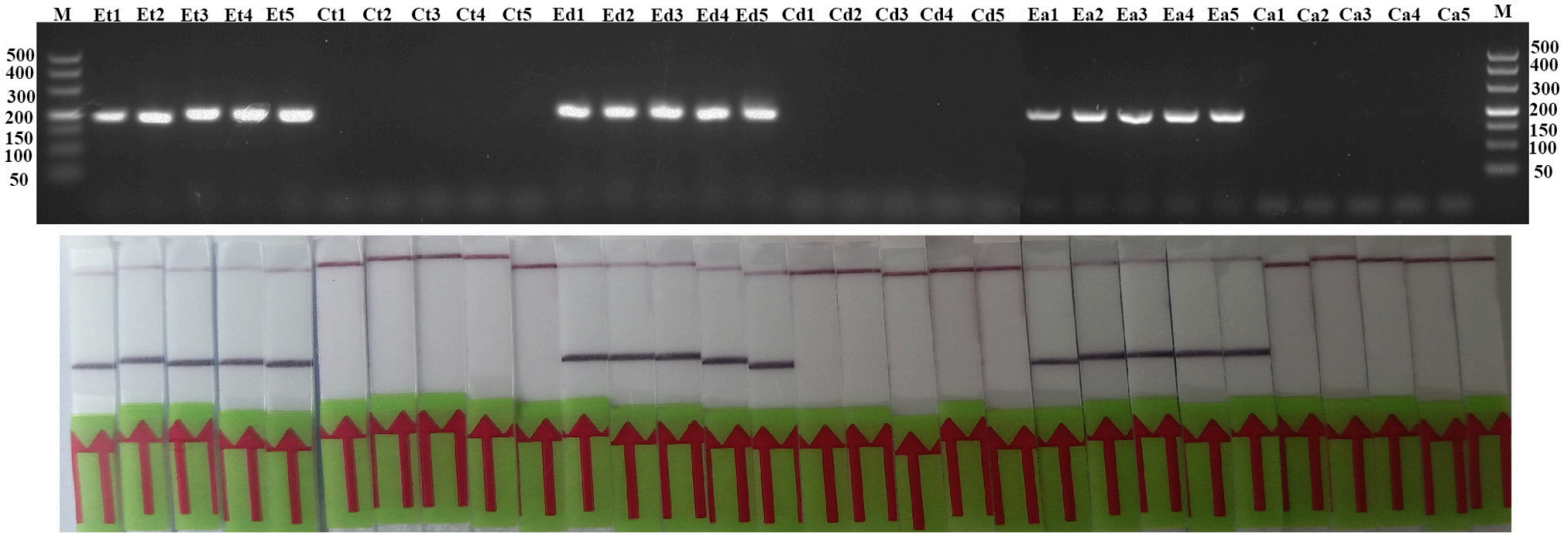
Evaluation of LF-RPA and conventional PCR assay for experimentally infected samples. Et, tongue tissues from experimentally infected group; Ct, tongue tissues from control group; Ed, diaphragm tissues from experimentally infected group; Cd, diaphragm tissues from control group; Ea, abdominal muscle tissues from experimentally infected group; Ca, abdominal muscle tissues from control group.

The tissues of experimentally infected mice were used to assess the performance of RPA assay in comparison with conventional PCR assay. Before DNA extraction, all experimentally infected tissues (tongue, diaphragm, and abdominal muscle) were compressed and examined by microscope and the results showed that all infected tissues contained the infective larvae and the highest larvae burden was detected in the diaphragm. Tissues from experimentally infected mice can be detected by both LF-RPA assay and conventional PCR assay and there were no positive samples from naïve control group (Figure 6).

## Discussion

At present, China is one of several countries with the highest number of human cases of trichinellosis in the world (Cui and Wang, 2011; Bai et al., 2017). During 1964-2011, approximately 600 outbreaks of human trichinellosis were reported in China, involving 38797 individuals and claiming the life of 336 persons (Cui and Wang, 2011; Bai et al., 2017). The high prevalence of trichinellosis in China is related to eating habits, pig breeding and lack of effective surveillance system. In addition, there has not been mandatory test for *Trichinella* spp. larvae in meat used for human consumption except pork in China (Bai et al., 2017). Thus, an accurate, sensitive, and convenient method for the detection of *Trichinella* infection in the meat is needed in order to achieve an effective parasite control.

Recently, the RPA assay technique has been successfully applied for the detection of schistosomiasis (Rosser et al., 2015; Sun et al., 2016; Xing et al., 2017), cryptosporidiosis (Wu et al., 2016), malaria (Kersting et al., 2014), leishmaniosis (Mondal et al., 2016; Saldarriaga et al., 2016), theileriosis (Yin et al., 2017), and fascioliosis (Cabada et al., 2017). Compared with conventional PCR, RPA assay has exhibited several advantages, such as high sensitivity, rapid detection time, convenient operation, and less requirement for specialized equipment. Additionally, the RPA amplification products can be detected by the naked eye with a lateral flow strip. All these features make LF-RPA well-suited for field application (James and Macdonald, 2015; Daher et al., 2016).

The LF-RPA assay developed in this study has a high species specificity that could detect all known *Trichinella* species and genotypes. In addition, there was no cross-reactivity with DNA from any other parasites and related hosts of *Trichinella* under the used experimental conditions, suggesting that this LF-RPA has good specificity. Further studies should focus on verifying potential cross-reactivity with DNA from other swine pathogens using the LF-RPA method developed herein.

The developed RPA assay was also highly sensitive and could detect down to 0.001 larvae per reaction, which was almost 10-folds more sensitive than the conventional PCR assay. Furthermore, the RPA amplification products can be detected using a lateral flow strip or agarose gel electrophoresis. For detection by the lateral flow strip, it only requires 5 min and is more visible than that detected by agarose gel electrophoresis. Compared with other diagnostic tests used to detect *Trichinella* infection, the time required of the RPA amplification is the shortest. Usually, conventional PCR, real-time PCR and LAMP require more than 1 h to complete in a well-equipped laboratory (Gottstein et al., 2009; Shimoni and Froom, 2015). Moreover, the developed LF-RPA can work well in a wide range of temperature (25–45°C), and can be performed in a water-bath. Furthermore, RPA assay showed a degree of tolerance to inhibitors in the muscle, suggesting that LF-RPA is more stable than the conventional PCR systems, which would facilitate large-scale investigation of the prevalence of *Trichinella*.

The validity of the LF-RPA assay was further analyzed by using tissue of mice that had been experimentally infected with *T. spiralis*, compared it with microscopy and conventional PCR. Our results showed that the developed LF-RPA exhibited 100% sensitivity and specificity under the used experimental conditions. Therefore, the RPA assay is more suitable for usage under field conditions, especially in resource-limited settings.

In conclusion, a LF-RPA assay was successfully developed for the convenient, rapid, specific and sensitive detection of all known *Trichinella* species and genotypes. The developed LF-RPA assay does not require any expensive instrumentation for DNA amplification and has an easy read-out system in comparison with conventional PCR. These results suggest that the developed LF-RPA can provide an alternative method for meat screening and point-of-care diagnosis in *Trichinella* spp. infection in food animals.

## Ethics Statement

The study was approved by the Animal Administration and Ethics Committee of Lanzhou Veterinary Research Institute, Chinese Academy of Agricultural Sciences (Permit No. LVRIAEC-2013-006). All experimental mice were handled in strict accordance with the Guidelines of the People's Republic of China and Animal Ethics Procedures.

## Author Contributions

B-QF and W-ZJ designed this study and critically revised the manuscript. T-TL, J-LW, and N-ZZ performed the experiments, analyzed data and drafted the manuscript. W-HL, H-BY, and LL participated in manuscript revision. All the authors read and approved the final manuscript.

### Conflict of Interest Statement

The authors declare that the research was conducted in the absence of any commercial or financial relationships that could be construed as a potential conflict of interest.

## Supplementary Material

Figure S1 Sensitivities of the LF-RPA assay and conventional PCR assay for detecting isolated genomic DNA from *T. pseudospiralis*. Ten-fold serial dilutions of isolated *T. pseudospiralis* genomic DNA (1 ng/reaction to 10 fg/reaction) were evaluated by LF-RPA **(A)** and conventional PCR assay using agarose gel electrophoresis **(B)**. Lower limit of detection can be seen at 100 fg and 1 pg of *T. pseudospiralis* DNA by LF-RPA assay and conventional PCR assay, respectively. NC, Negative Control.

Figure S2 Sensitivities of the LF-RPA assay and conventional PCR assay for detecting *T. pseudospiralis* DNA extracted from swine muscle. One-gram from each muscle sample was spiked with one *T. pseudospiralis* larvae and 10-fold serial dilutions of this DNA were evaluated by LF-RPA **(A)** and conventional PCR assay detected by agarose gel electrophoresis **(B)**. Lower limit of detection can be seen at 1:100 and 1: 10 dilutions of this DNA by LF-RPA assay and conventional PCR assay, respectively. NC, Negative Control.

Figure S3 Testing the effect of inhibitors on the LF-RPA assay and conventional PCR assay for detecting the DNA from *T. pseudospiralis*. Different percentage of muscle penetrating fluid in the reaction mixture were evaluated by LF-RPA assay **(A)** and conventional PCR assay **(B)**. LF-RPA assay performed better than conventional PCR assay in the presence of potential inhibitors of the amplification reaction. NC, Negative Control.
