# Supplementary figures and images for: Rapid and Visual Detection of Trichinella Spp. Using a Lateral Flow Strip-Based Recombinase Polymerase Amplification (LF-RPA) Assay
Source: Front Cell Infect Microbiol. 2019 Jan 21;9:1. doi: 10.3389/fcimb.2019.00001 (PMC6348712; doi:10.3389/fcimb.2019.00001)

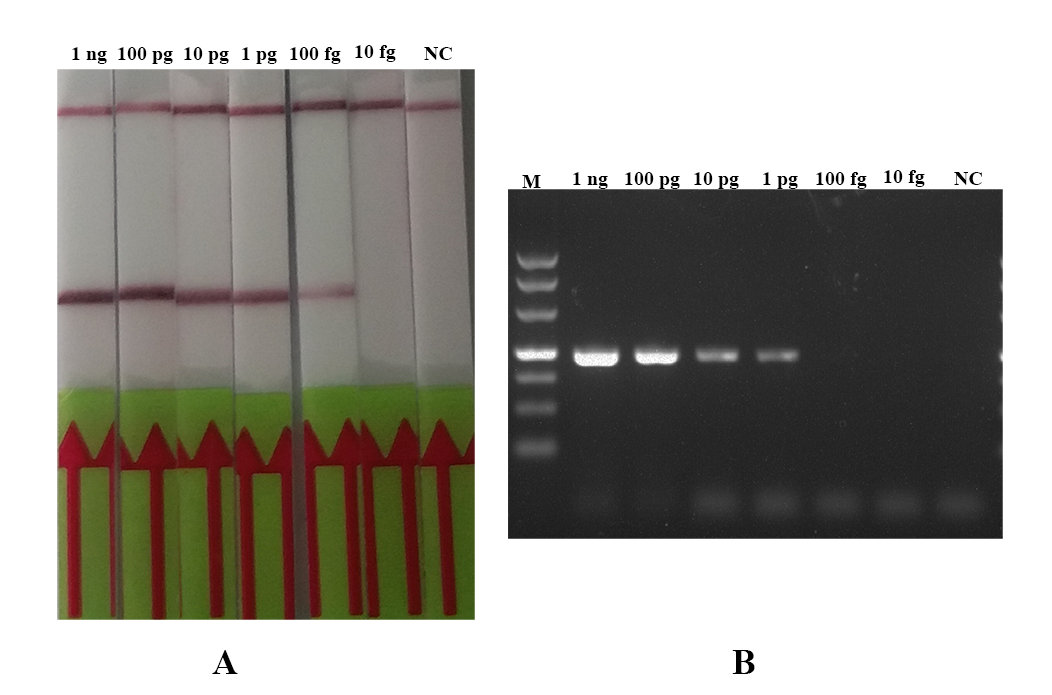

Supplement: Figure S1 — Sensitivities of the LF-RPA assay and conventional PCR assay for detecting isolated genomic DNA from T. pseudospiralis. Ten-fold serial dilutions of isolated T. pseudospiralis genomic DNA (1 ng/reaction to 10 fg/reaction) were evaluated by LF-RPA (A) and conventional PCR assay using agarose gel electrophoresis (B). Lower limit of detection can be seen at 100 fg and 1 pg of T. pseudospiralis DNA by LF-RPA assay and conventional PCR assay, respectively. NC, Negative Control. [file Image_1.TIF]

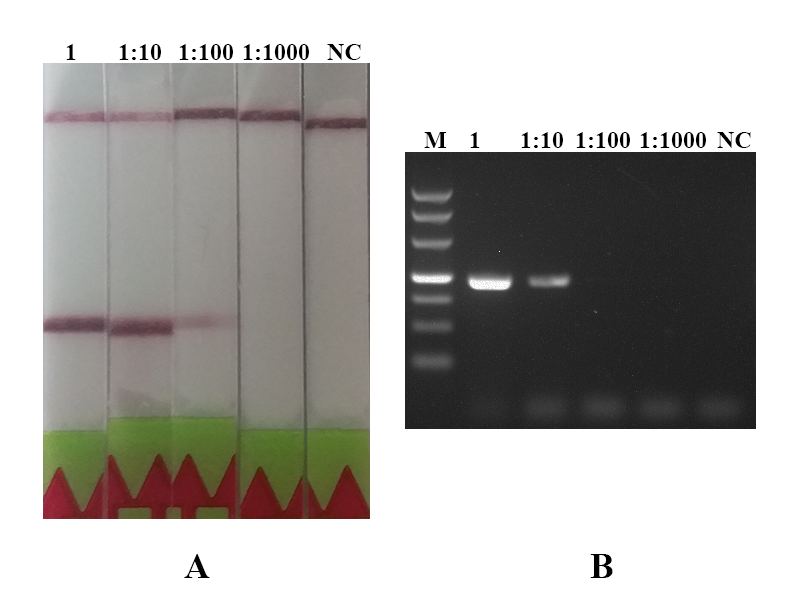

Supplement: Figure S2 — Sensitivities of the LF-RPA assay and conventional PCR assay for detecting T. pseudospiralis DNA extracted from swine muscle. One-gram from each muscle sample was spiked with one T. pseudospiralis larvae and 10-fold serial dilutions of this DNA were evaluated by LF-RPA (A) and conventional PCR assay detected by agarose gel electrophoresis (B). Lower limit of detection can be seen at 1:100 and 1: 10 dilutions of this DNA by LF-RPA assay and conventional PCR assay, respectively. NC, Negative Control. [file Image_2.TIF]

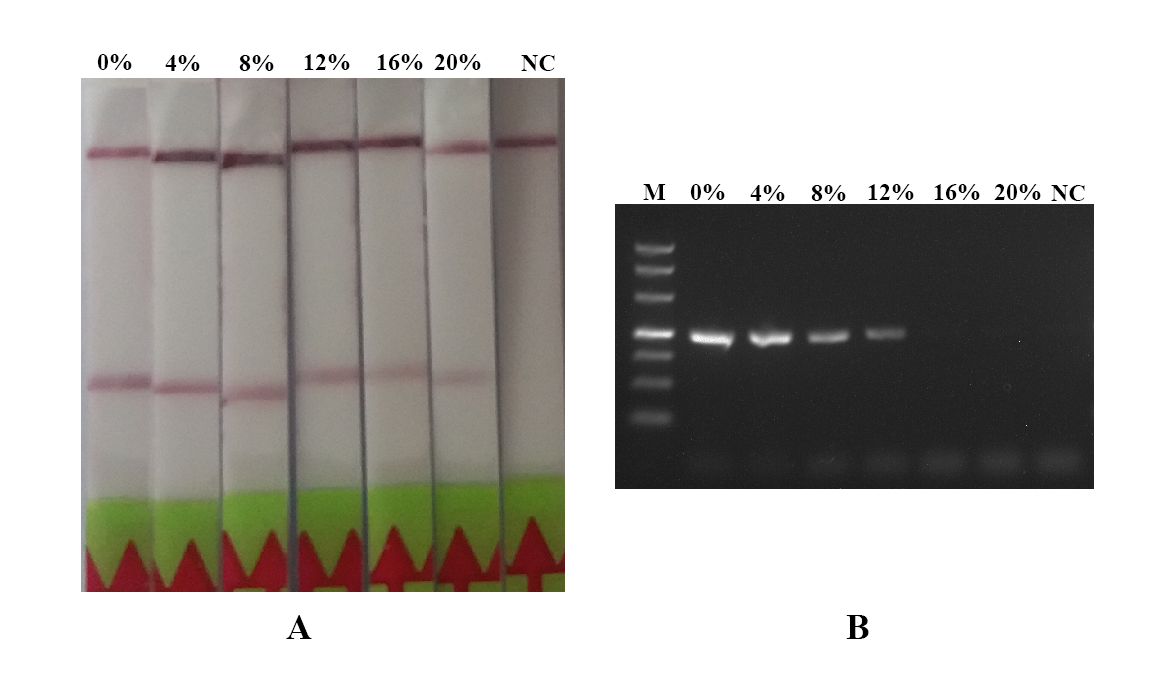

Supplement: Figure S3 — Testing the effect of inhibitors on the LF-RPA assay and conventional PCR assay for detecting the DNA from T. pseudospiralis. Different percentage of muscle penetrating fluid in the reaction mixture were evaluated by LF-RPA assay (A) and conventional PCR assay (B). LF-RPA assay performed better than conventional PCR assay in the presence of potential inhibitors of the amplification reaction. NC, Negative Control. [file Image_3.TIF]
